# Suprachoroidal Triamcinolone Injection for the Management of Chronic Uveitis‐Associated Ocular Hypotony

**DOI:** 10.1155/crop/2476023

**Published:** 2026-06-27

**Authors:** Dan Spiegelman, Isabella Song, Erin Sieck

**Affiliations:** ^1^ Washington University in St. Louis School of Medicine, St. Louis, Missouri, USA, wustl.edu; ^2^ John F. Hardesty, MD Department of Ophthalmology and Visual Sciences, Washington University in St. Louis, St. Louis, Missouri, USA, wustl.edu

**Keywords:** ocular hypotony, steroid therapy, suprachoroidal triamcinolone, uveitis

## Abstract

**Purpose:**

This study is aimed at describing the use of suprachoroidal triamcinolone injection for the treatment of refractory uveitis‐associated ocular hypotony presumed secondary to ciliary body shutdown.

**Methods:**

We report a case of a 58‐year‐old woman with a 26‐year history of idiopathic, noninfectious bilateral panuveitis complicated by recurrent cystoid macular edema and a steroid‐induced ocular hypertension. After treatment with an intravitreal dexamethasone implant and an Ahmed glaucoma valve, she developed persistent hypotony in the right eye with intraocular pressure (IOP) of 2–4 mmHg despite controlled inflammation. Evaluation with gonioscopy and ultrasound biomicroscopy revealed no cyclodialysis cleft or cyclitic membranes. Prior treatments, including topical steroids, repeat dexamethasone implant, Ahmed valve revision, and serial anterior chamber viscoelastic injections, produced only transient improvement. Suprachoroidal triamcinolone (40 mg) was administered.

**Results:**

Before injection, IOP ranged from 2 to 6 mmHg. Following treatment, IOP remained ≥ 6 mmHg for approximately 2 months. Best corrected visual acuity improved from 20/80 to 20/50, and additional viscoelastic injections were not required during this period. Numerical hypotony recurred 8 months later.

**Conclusion:**

Suprachoroidal triamcinolone may represent a targeted treatment for refractory uveitis‐associated hypotony when ciliary body dysfunction is suspected and other steroid delivery routes are limited. Further study is needed to evaluate durability and broader applicability.

## 1. Introduction

Ocular hypotony is a condition that typically occurs when aqueous production is inadequate to maintain a physiologic intraocular pressure (IOP) [1–3]. Although clinical hypotony is defined as low IOP with related anatomical changes, there is no agreed‐upon definition of numerical hypotony, as different studies use cutoffs between 5 [4] and 9 mmHg [5]. While choroidal folds are the most widely recognized complication of hypotony, other common exam features include a shallow anterior chamber (AC), Descemet′s membrane folds, and choroidal effusions [1].

Although hypotony is uncommon in the general population, it affects between 1.2% and 10.0% of patients with uveitis [2] and is associated with a high risk of vision loss [5]. Multiple mechanisms can lead to an inability to maintain a physiologic IOP in uveitis, including (1) hyposecretion of aqueous humor from the ciliary body secondary to active inflammation, (2) increased outflow due to ciliary body detachment, (3) atrophy of the ciliary processes due to chronic inflammation, (4) increased uveoscleral outflow, (5) hyposecretion of the ciliary body due to traction by cyclitic membranes, and most likely (6) a combination of all of the above [2, 6]. Hypotony can also be seen after glaucoma surgeries or lasers [2]. Current treatments for uveitis‐induced hypotony focus on controlling inflammation, relieving any tractional forces on the ciliary body, and inducing a steroid hypertensive response using systemic corticosteroids, topical steroids, and periocular or intravitreal steroid injections [6, 7]. However, despite these interventions, hypotony can be resistant to common therapies.

Here, we present a case of a 58‐year‐old patient with chronic uveitis complicated by recurrent cystoid macular edema (CME) who developed hypotony in both eyes despite controlled inflammation with the absence of AC or vitreous cell, thought to be secondary to ciliary body shutdown and aqueous hyposecretion. Her hypotony failed to respond to topical steroids but ultimately stabilized following suprachoroidal triamcinolone injection.

## 2. Case Presentation

Our patient is a 58‐year‐old female who presented with a 26‐year history of idiopathic recurrent nongranulomatous, noninfectious bilateral panuveitis with recurrent CME (Figure S1). Her medical history was significant for migraine, metabolic dysfunction‐associated steatotic liver disease (MASLD), cholelithiasis, and mast cell activation syndrome. She experienced multiple adverse reactions to topical corticosteroids, primarily ocular surface irritation. Given the irritation, she also was treated with routes of steroid administration including subtenon, intravitreal, and systemic. With her initial treatments, she developed an ocular hypertensive response with a maximum pressure > 40 mmHg in the right eye (OD) outside of our system. She declined systemic immunosuppression due to concern for side effects.

Upon presentation to our institution, she had persistent CME in both eyes impacting visual acuity. Given her history of steroid response, a dexamethasone intravitreal implant was implanted OD to treat the CME with a concurrent Ahmed glaucoma drain to blunt any pressure spikes. The intravitreal implant was successful at resolving her CME, and her IOP remained controlled through the immediate postoperative course. Her IOP was 9 mmHg 3 months after surgery, and she had no clinical signs of hypotony including normal optical coherence tomography (OCT) not demonstrating hypotony maculopathy.

Despite no change in management, 4 months postoperatively, her IOP declined to 2 mmHg OD. Despite the addition of topical difluprednate ranging from twice to four times daily administration, her IOP remained at 4 mmHg 8 months after the Ahmed implant was placed. A second dexamethasone implant was placed at this time intended to induce a steroid response. Despite these interventions, her IOP remained 2–4 mmHg. This numerical hypotony persisted for another 12 months with preserved visual acuity. Twenty months post‐Ahmed implantation, she had progressive signs of clinical hypotony, with macular folds and a temporal and nasal choroidal effusion. It was decided to undergo surgical revision of her Ahmed implant and lens exchange for intralenticular inclusions of her single‐piece acrylic intraocular lens. Surgical intervention of her hypotony was delayed as she had preserved visual function and the patient′s desire to limit surgical interventions with maintained vision. When she developed a multifactorial decrease in vision due to new‐onset choroidal folds and intralenticular inclusions, a combined surgical approach was planned.

The Ahmed valve was tied with 6‐0 prolene and was watertight in the operating room. Intraoperatively, there was poor zonular support for a sulcus lens. A scleral fixated lens was not selected due to the concern for postoperative hypotony due to scleral tunnel peritubular flow. Thus, an ACIOL was chosen. The single‐piece lens was exchanged for a MTA4UO (Alcon) lens. Her pressure transiently improved on the right eye 1 month postoperatively to a maximum pressure of 12 mmHg. By postoperative Month 2 from the Ahmed revision, the pressure returned to 4 mmHg.

Gonioscopy was normal. Ultrasound biomicroscopy (UBM) did not demonstrate cyclodialysis clefts or cyclitic membranes (Figure S2). Given persistent hypotony, prolonged inflammation, and without findings suggestive of another cause, her hypotony was favored to be secondary to ciliary body shutdown.

At this time, another round of topical steroids was tried without improvement in IOP. An intravitreal dexamethasone implant was contraindicated due to the AC lens. She deferred a subtenon′s depot of steroids due to systemic side effects with subtenon′s injections prior. With limited options, the AC was reformed with hyaluronidase‐based viscoelastic, Healon GV (Johnson & Johnson), to a pressure of > 10 mmHg. With the increased pressure, her visual acuity improved from best corrected visual acuity (BCVA) 20/80 preinjection to BCVA 20/50 with subjectively improved functionality. She had eight AC fills over 12 months. However, the effects of the viscoelastic injection consistently wore off within weeks with pressures returning to ≤ 8 mmHg.

During the series of AC fills, CME worsened OD. She had limited options for therapy. Because of previous severe side effects from prior subtenon′s and intravitreal steroid administrations, the decision was made to inject triamcinolone into the suprachoroidal space as a potentially more favorable route. A total of 40 mg of preservative‐free triamcinolone at a concentration of 40 mg/mL was injected. Prior to injection, her IOP OD ranged from 2 to 6 mmHg. Her IOP for 2 months after the suprachoroidal injection OD repeatedly measured 6–7 mmHg, with preserved visual acuity and improvement in CME. Eight months following suprachoroidal triamcinolone administration, she developed numerical hypotony OD at 4 mmHg and visual acuity worsened again. The AC fills of viscoelastic were resumed with eventual plan for another injection of suprachoroidal triamcinolone. The clinical timeline is shown in Table 1.

**Table 1:** Clinical timeline.

**Time (months)**	**Clinical information and interventions**
0	○ Dexamethasone intravitreal implant + Ahmed valve OD for CME complicated by self‐resolving vitreous hemorrhage
1	○ CME resolved, IOP ↓ to 7 mmHg OD
4	○ Hypotony with IOP ↓ to 2 mmHg OD
	○ Difluprednate TID showed IOP ↑ to 9 mmHg OD, frequency reduced to BID due to patient intolerance
8	○ Hypotony with IOP ↓ to 4 mmHg OD
	○ No IOP response to increased steroid frequency or dexamethasone intravitreal implants
23	○ Clinical hypotony develops due to persistent low IOP OD: developed macular folds, choroidal effusions, and visual impairment
	○ Ahmed valve tied off
	○ Lens exchange performed OD due to intralenticular opacification
24–25	○ Transient IOP ↑ to 12 mmHg OD, but later reverted to 5 mmHg OD
	○ UBM and gonioscopy negative; ciliary body shutdown suspected
39–48	○ With limited therapeutic options, seven hyaluronidase‐based AC fills with IOP ↑ to > 10 mmHg OD and transiently improved visual acuity. IOP returned to < 8 mmHg OD after 1–2 weeks of each fill
	○ CME worsened OD
	○ Treatment options limited: poor response to topical drops, side effects to steroids
49	○ Suprachoroidal triamcinolone injection OD with IOP ↑ to ≥ 6 mmHg OD
	○ CME improved
51	○ IOP ↓ to 5 mmHg OD
	○ AC fill with transient IOP ↑ to > 20 mmHg OD, which was decreased with removal of viscoelastic to 18 mmHg OD
55	Stable IOP ≥6 mmHg OD
	○ No worsening macular folds or choroidal effusions
	○ Stable visual acuity

Abbreviations: BID, twice a day; CME, cystoid macular edema; IOP, intraocular pressure; OD, right eye; TID, three times a day; UBM, ultrasound biomicroscopy.

## 3. Discussion

Ocular hypotony is an uncommon sequela of chronic uncontrolled uveitis. It likely occurs due to a combination of mechanisms causing reduced aqueous humor production from an inflamed and damaged ciliary body [2]. Most treatment options in the literature focus on a variety of steroid administrations to reduce inflammation and create a hypertensive steroid response to elevate pressure [3, 8]. There are a few conservative measures described using sympathomimetics or cycloplegics to target the position of the ciliary body. Surgically, there are approaches using viscoelastics, balanced salt solution, or silicone oil to reduce aqueous outflow and add volume to increase the IOP [1].

We present a case of recalcitrant uveitis‐associated ocular hypotony that failed conservative therapies. Our patient demonstrated historical elevations in IOP due to steroid use. “Steroid response” describes patients that develop an ocular hypertensive response to steroid administration. Steroids increase IOP by increasing resistance to flow at the trabecular meshwork, typically weeks after initiation, though this can also happen acutely [9]. Studies of the trabecular meshwork have shown an increase in deposition of glycosaminoglycans and reduced activity of matrix metalloproteinases when steroids are used. As a result, this increase in extracellular matrix deposition reduces flow of aqueous humor across the trabecular meshwork and increases IOP [10].

Topical and intraocular routes of administration for steroids in particular are associated with steroid response. For instance, approximately 18%–36% of patients treated with topical corticosteroids are classified as steroid responders [11]. Intravitreal corticosteroid injections are also associated with increased IOP:The SCORE study performed on patients with macular edema due to retinal vein occlusion reported a 45% cumulative incidence of IOP elevation > 10 mmHg from baseline at 36 months for 4 mg intravitreal triamcinolone, and 9% for 1 mg intravitreal triamcinolone [12]. Similarly, intravitreal steroid implants are associated with dose‐dependent elevations, with a rate of IOP elevation > 10 mmHg in 65% of uveitic patients receiving a surgical 0.59‐mg fluocinolone implant after 2 years [13], and 26% in uveitic patients receiving intravitreal dexamethasone implants after 24 weeks [14]. Periocular injections of steroids are also associated with IOP elevation, though to a lesser degree than intravitreal implants [14].

In contrast to the rates of IOP increase in previous studies, only 11.5% of uveitic patients treated with suprachoroidal triamcinolone injection in the PEACHTREE Trial experienced an IOP increase [15]. This may be due to the localized distribution of the steroid in the suprachoroidal space, adjacent to the ciliary body but not the trabecular meshwork.

In our case, our patient stopped having a traditional ocular hypertensive response to topical difluprednate or intravitreal dexamethasone. We postulate that ciliary body shutdown and reduced aqueous humor formation contributed to her hypotony. Thus, increasing resistance to outflow with topical steroid use may have been inadequate to increase her IOP. The use of the suprachoroidal steroid injection localized steroid therapy to her ciliary body. As a result, her eye likely began making more aqueous humor, which increased her pressure and reduced signs of clinical hypotony. However, it is difficult to exclude alternative explanations such as prior interventions or natural fluctuations in IOP.

A limitation to this study is due to the difficulty in distinguishing inflammatory hyposecretion from surgery‐related mechanisms of hypotony. We excluded other etiologies of her hypotony with normal gonioscopy and UBM. Although the number of surgeries she had made it difficult to attribute her hypotony to hyposecretion of the ciliary body, it seems the most likely cause.

Here, we present a case of uveitis‐associated hypotony that responded favorably to a suprachoroidal steroid injection. The use of suprachoroidal triamcinolone in this context may represent a new avenue for treating hypotony refractory to standard therapies. Further observations are required to determine if there is benefit to this type of injection.

## Author Contributions

D.S. and I.S. were involved in data collection and manuscript drafting. E.S. was responsible for project conceptualization, supervision, and manuscript revision. All authors attest that they meet the current ICMJE criteria for authorship.

## Funding

No funding was received for this manuscript.

## Disclosure

After conducting a literature review on June 28, 2025, utilizing PubMed, Google Scholar, Scopus, and Embase, using the keywords “uveitis,” “ocular hypotony,” and “suprachoroidal triamcinolone,” we did not find any prior reports describing the use of suprachoroidal triamcinolone injection for the treatment of uveitis‐associated hypotony.

## Ethics Statement

This retrospective review of patient data did not require ethical approval in accordance with local and national guidelines. Written informed consent was obtained from the patient for publication of the details of their medical case and any accompanying images.

## Conflicts of Interest

The authors declare no conflicts of interest.

## Supporting information


**Supporting Information** Additional supporting information can be found online in the Supporting Information section. Figure S1: Available optical coherence tomography (OCT) images demonstrating cystic macular edema and choroidal folds during the patient′s course. Available central macular thickness (CMT) values are noted above each representative image. Figure S2: Ultrasound biomicroscopy (UBM) images of (A) the right eye over the Ahmed valve, without peritubular flow, (B) the right eye without cyclodialysis clefts or cyclitic membranes, and (C) the left eye without cyclodialysis clefts or cyclitic membranes.

## Data Availability

This study did not generate any datasets. All information presented in this case report is contained within the article and anonymized to protect patient privacy. Further enquiries can be directed to the corresponding author.
