# Supplementary material for: Suprachoroidal Triamcinolone Injection for the Management of Chronic Uveitis‐Associated Ocular Hypotony
Source: Case Rep Ophthalmol Med. 2026 Jun 27;2026:2476023. doi: 10.1155/crop/2476023 (PMC13309794; doi:10.1155/crop/2476023)
Supplement: Supplementary file 1 — Supporting Information Additional supporting information can be found online in the Supporting Information section. Figure S1: Available optical coherence tomography (OCT) images demonstrating cystic macular edema and choroidal folds during the patient′s course. Available central macular thickness (CMT) values are noted above each representative image. Figure S2: Ultrasound biomicroscopy (UBM) images of (A) the right eye over the Ahmed valve, without peritubular flow, (B) the right eye without cyclodialysis clefts or cyclitic membranes, and (C) the left eye without cyclodialysis clefts or cyclitic membranes. [file CROP-2026-2476023-s001.pdf]

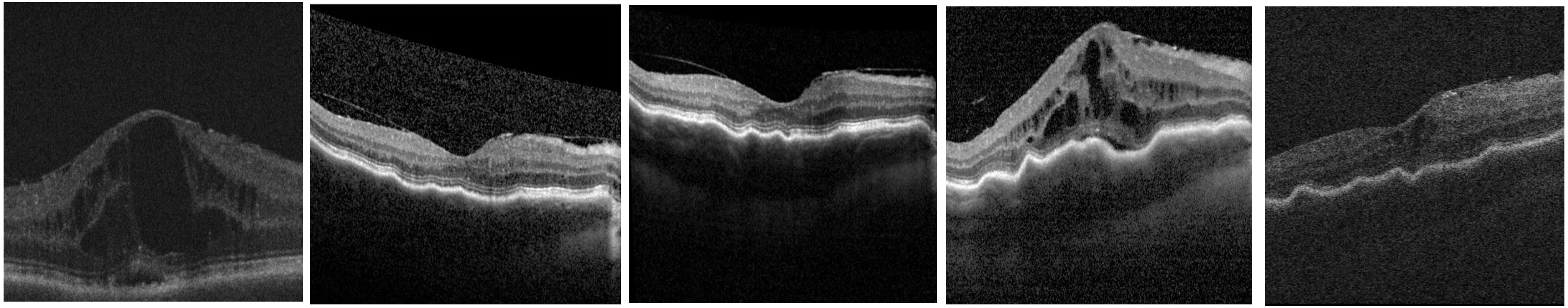

Time	Month 2	Month 6	Month 8	Month 32	Month 39
CMT	564 µm	-	226 µm	908 µm	362 µm

Supplemental Figure 1: Available optical coherence tomography (OCT) images demonstrating cystic macular edema and choroidal folds during the patient's course. Available central macular thickness (CMT) values are noted above each representative image.

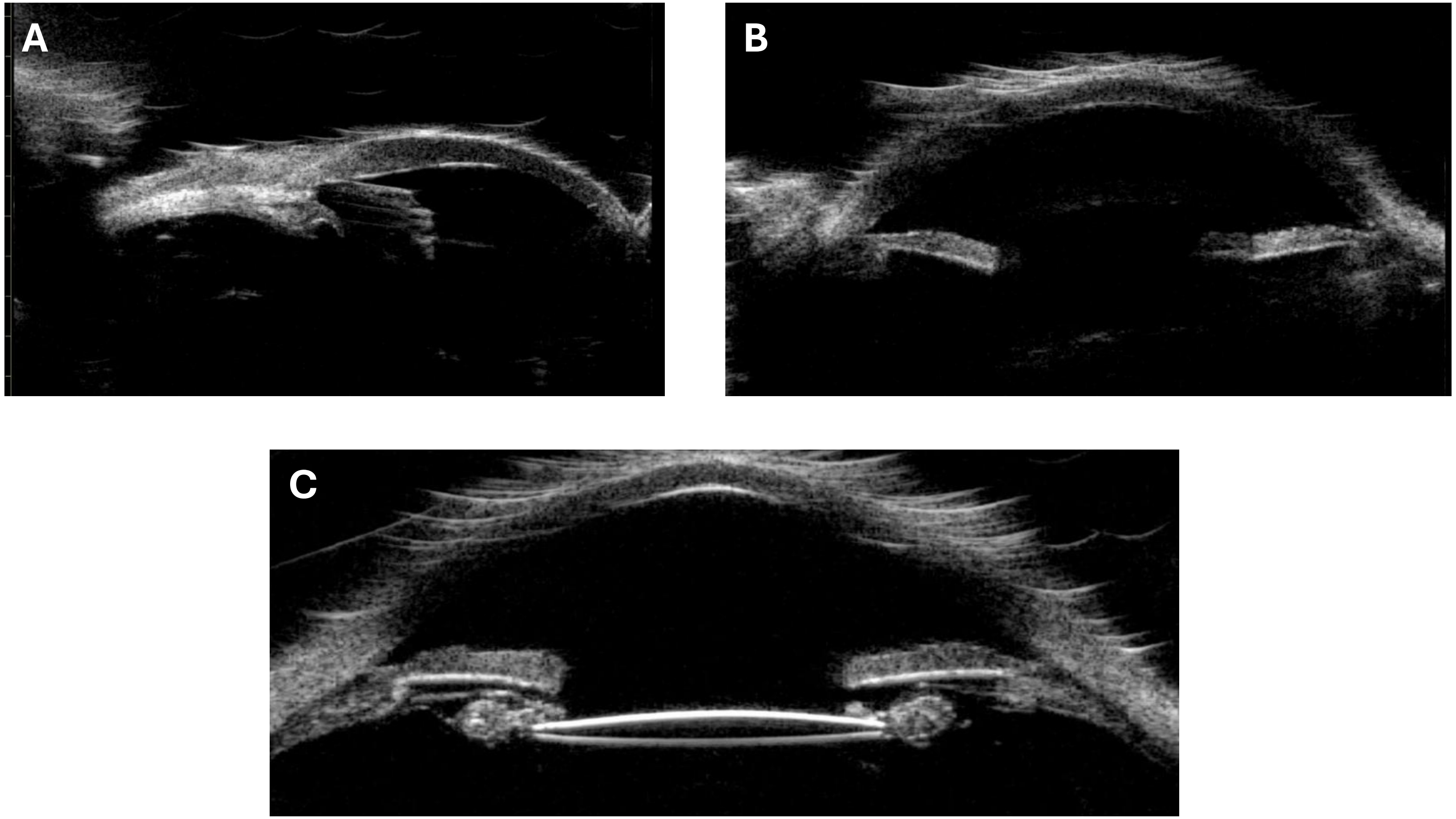

Supplemental Figure 2: Ultrasound biomicroscopy (UBM) images of A) the right eye over the Ahmed valve, without peritubular flow, B) the right eye without cyclodialysis clefts or cyclitic membranes, and C) the left eye without cyclodialysis clefts or cyclitic membranes.
